# Self-focused attention, cost/probability bias, and avoidance behavior mediate the relationship between trait mindfulness and social anxiety: A cross-sectional study

**DOI:** 10.3389/fpsyg.2022.942801

**Published:** 2022-08-08

**Authors:** Shota Noda, Kentaro Shirotsuki, Satoko Sasagawa

**Affiliations:** ^1^Graduate School of Human and Social Sciences, Musashino University, Tokyo, Japan; ^2^Japan Society for the Promotion of Science, Tokyo, Japan; ^3^Faculty of Human Sciences, Musashino University, Tokyo, Japan; ^4^Faculty of Psychology, Mejiro University, Tokyo, Japan

**Keywords:** anxiety, mindfulness, attention, cross-sectional studies, Japan

## Abstract

Self-focused attention, cost/probability bias, and avoidance behavior are maintaining factors for social anxiety. In particular, cost bias and avoidance behavior predict social anxiety. It has been shown that the enhancement of trait mindfulness improves these maintaining factors. This study examines the relationships among trait mindfulness, self-focused attention, cost/probability bias, avoidance behavior, and social anxiety, and clarifies whether they mediate the relationship between trait mindfulness and social anxiety. A cross-sectional design was used to examine the relationships among these variables. Participants were recruited from three universities in Japan (January 2019–December 2019). Undergraduate students (*N* = 367) completed a set of self-report measures assessing trait mindfulness, self-focused attention, cost/probability bias, avoidance behavior, and social anxiety. Results of path analyses revealed that the hypothesized model’s goodness-of-fit indices had high values. Trait mindfulness showed a direct negative association with self-focused attention, cost/probability bias, avoidance behavior, and social anxiety. Moreover, trait mindfulness was negatively associated with social anxiety *via* self-focused attention, cost/probability bias, and avoidance behavior. These findings indicate that mindfulness plays an important role in social anxiety and provide impetus for future research involving clinical studies of mindfulness-based interventions for social anxiety.

## Introduction

Social anxiety disorder (SAD) is relatively common, with a lifetime prevalence of 12.1% in the United States (Kessler et al., 2005) and 1.4% in Japan (Ishikawa et al., 2016); the mean age of onset is 15.1–19.3 years (Grant et al., 2005; Shindo et al., 2006; Acarturk et al., 2008). SAD has a high comorbidity rate with other mental health disorders, such as other anxiety disorders, mood disorders, and substance-related disorders (Magee et al., 1996; Acarturk et al., 2008; Ohayon and Schatzberg, 2010). In addition, it has been associated with significant impairment in social, educational, and occupational functioning, and lower quality of life (Stein and Kean, 2000; Acarturk et al., 2008).

According to Ruscio (2010), SAD exists on a continuum along with less severe social anxiety symptoms. McNeil (2010) also suggested that social anxiety symptoms, like those of other phobic disorders, exist along a continuum in the general population. There are no significant differences in the levels of psychological and physiological characteristics between SAD patients and individuals with high social anxiety symptoms (Turner et al., 1986), and the two groups share several similar features, including somatic and cognitive responses (Turner et al., 1990). Therefore, SAD studies should examine not only SAD patients but also individuals with high social anxiety whose symptoms are not considered sufficiently severe to warrant a diagnosis.

According to the cognitive behavioral models of SAD (Clark and Wells, 1995; Rapee and Heimberg, 1997; Hofmann and Otto, 2008), self-focused attention, cost/probability bias, and avoidance behavior are maintaining and exacerbating factors of social anxiety. Self-focused attention refers to the perception of internal self-related information, such as body state, thoughts, feelings, and behaviors in threatening social situations (Bögels et al., 1996; Noda et al., 2021a). Individuals with high self-focused attention have a higher degree of social anxiety symptoms than those with low self-focused attention (Noda et al., 2021a). Furthermore, heightened self-focused attention increases negative cognitions such as cost/probability bias, and contributes to the exacerbation of social anxiety and avoidance behavior (Clark and Wells, 1995; Rapee and Heimberg, 1997; Hofmann and Otto, 2008).

Cost/probability bias refers to specific negative cognitions in SAD. Cost bias is the overestimation of costs associated with negative social events, and probability bias is characterized by exaggerated estimates of the occurrence of negative events (Foa et al., 1996). Cost/probability bias is moderately to strongly correlated with social anxiety symptoms (Ito et al., 2019). In particular, cost bias has a moderate impact on social anxiety (Shirotsuki et al., 2010; Noda et al., 2017b) and has been shown to increase avoidance behavior (Hofmann and Otto, 2008; Shirotsuki et al., 2010).

Avoidance behavior, which is a common feature of anxiety disorders, describes actions taken to avoid certain situations in which anxiety occurs. When faced with threatening social situations, individuals with high social anxiety adopt avoidance behavior to relieve anxiety (Hofmann and Otto, 2008). While avoidance behavior temporarily relieves anxiety, it also increases social anxiety in the long term (Clark and Wells, 1995; Hofmann and Otto, 2008). Avoidance behavior is strongly correlated with (Noda et al., 2017a,2018a) and increases social anxiety (Okajima et al., 2009).

Cognitive behavioral therapy (CBT) programs for social anxiety target not only social anxiety but also its maintaining factors. Previous studies suggest the importance of reducing self-focused attention, cost/probability bias, and avoidance behavior as treatment strategies for social anxiety (Mattick and Peters, 1988; Foa et al., 1996; Hofmann, 2004; Bögels, 2006; McManus et al., 2008; Shirotsuki et al., 2014; Vriends et al., 2017). Therefore, it is necessary to examine a treatment model that includes the three aforementioned variables to understand the mechanism of social anxiety.

In recent years, mindfulness-based interventions (MBIs) and mindfulness and acceptance-based group therapy (MAGT) have demonstrated efficacy in improving social anxiety and its maintaining factors (Koszycki et al., 2007; Piet et al., 2010; Kocovski et al., 2013; Goldin et al., 2016; Desnoyers et al., 2017). Mindfulness is defined as “paying attention in a particular way—on purpose, in the present moment and non-judgmentally” (Kabat-Zinn, 1994, p. 4). Trait mindfulness is negatively correlated with social anxiety and its maintaining factors, such as self-focused attention, cost/probability bias, and avoidance behavior (Schmertz et al., 2012; Noda et al., 2017a,2018a,b,2021a). Kocovski et al. (2015) also indicated that trait mindfulness predicts improvement in social anxiety.

Furthermore, recent studies have revealed relationships among trait mindfulness, social anxiety, and maintaining factors of social anxiety as mechanisms of mindfulness for social anxiety. According to Noda et al. (2018b), trait mindfulness affects fear of evaluation from others and avoidance behavior *via* self-focused attention, resulting in reduced social anxiety. It has been reported that trait mindfulness influences social anxiety *via* cost/probability bias (Schmertz et al., 2012) and avoidance behavior (Noda et al., 2017a). Based on the above, it was considered that self-focused attention, cost/probability bias, and avoidance behavior mediate the relationship between trait mindfulness and social anxiety.

However, the complex relationships among trait mindfulness, self-focused attention, cost/probability bias, avoidance behavior, and social anxiety have not been previously clarified. By elucidating the connection between these factors, it is possible to clarify the mechanisms by which trait mindfulness affects social anxiety symptoms. Therefore, this study aimed to investigate the relationships among trait mindfulness, self-focused attention, cost/probability bias, avoidance behavior, and social anxiety. For this purpose, hypothetical models were constructed, and the validity of the models was examined.

According to Shindo et al. (2006), the mean onset age of SAD in Japan is 18.6 years. In Japan university students, the average score on the Liebowitz Social Anxiety Scale (LSAS; Asakura et al., 2002), a screening index for SAD, has been reported to be 61.20 points (Noda et al., 2017a), which exceeds the cut-off value of 44 points for clinical groups (Asakura et al., 2002). Therefore, university students have a high degree of social anxiety symptoms, and this is the most common age at which SAD symptoms first appear. As it seems useful to examine university students to understand the psychological characteristics of SAD, the sample for the present study comprised undergraduate students.

## A hypothetical model

Previous studies examining the mechanism of mindfulness have shown that trait mindfulness impacts clinical symptoms of anxiety and depression *via* their maintaining factors (Schmertz et al., 2012; Desrosiers et al., 2013; Noda et al., 2017a,2018a,b). In this study, we hypothesized that (1) trait mindfulness affects social anxiety directly and (2) trait mindfulness impacts social anxiety through self-focused attention, cost/probability bias, and avoidance behavior. Therefore, we constructed three hypothetical models with trait mindfulness as the independent variable, social anxiety as the dependent variable, and self-focused attention, cost/probability bias, and avoidance behavior as the mediator variables.

**FIGURE 1:**
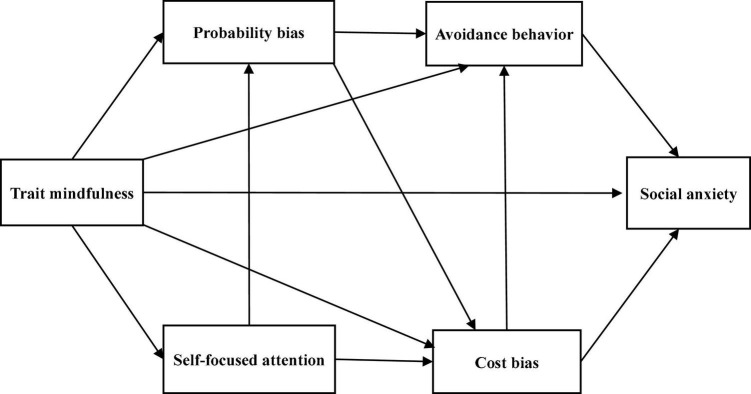
The hypothetical Model A.

**FIGURE 2:**
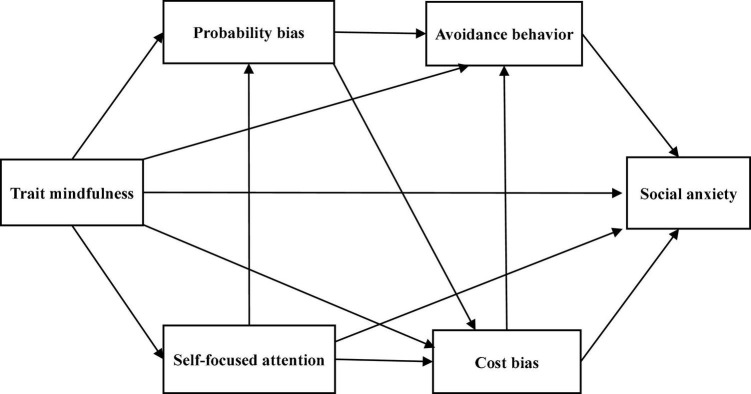
The hypothetical Model B.

**FIGURE 3:**
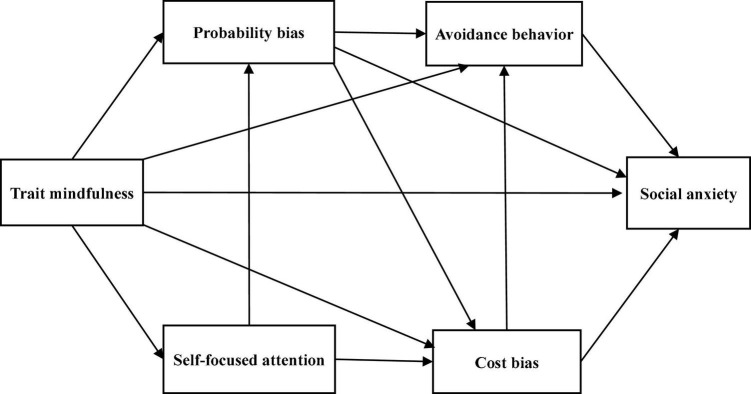
The hypothetical Model C.

Trait mindfulness has been found to affect each of the following factors directly: self-focused attention, cost bias, probability bias, avoidance behavior, and social anxiety (Schmertz et al., 2012; Noda et al., 2017a,2018b). A high state of self-focused attention implies sensitivity to internal self-related information in threatening social situations (Bögels et al., 1996; Noda et al., 2021a). Since mindfulness is an attitude of continuous awareness of external stimuli and internal experiences but not reacting to one’s own experiences (Cardaciotto et al., 2008), it may be negatively associated with self-focused attention. Cost bias and probability bias are cognitive biases characterized by negative estimates of future events, especially in performance situations (Foa et al., 1996). Since mindfulness is an attitude of paying attention to the present moment, not the past or future, and being receptive to oneself (Noda et al., 2022), it may be negatively associated with cost bias and probability bias. Mindfulness also enhances the ability to face fearful social situations (Barlow et al., 2011); thus, it may be negatively associated with avoidance behavior. In addition, mindfulness is an attitude of accepting oneself as one is, rather than concerning oneself with the evaluations of others (Kabat-Zinn, 1994). Therefore, it may be negatively associated with social anxiety. Based on the above, we assumed paths from trait mindfulness to self-focused attention, cost bias, probability bias, avoidance behavior, and social anxiety. Self-focused attention is an exacerbating factor of the cost/probability bias (Hofmann and Otto, 2008); therefore, paths from self-focused attention to cost bias and probability bias were hypothesized. Further, as cost and probability biases are aggravating factors of avoidance behavior (Hofmann and Otto, 2008), paths from cost bias and probability bias to avoidance behavior were set. Additionally, probability bias has been shown to have a positive effect on cost bias (Shirotsuki et al., 2010); therefore, we assumed a path from probability bias to cost bias. Finally, since cost bias and avoidance behavior increase social anxiety (Okajima et al., 2009; Shirotsuki et al., 2010; Noda et al., 2017b), paths from cost bias and avoidance behavior to social anxiety were hypothesized. Based on the above hypotheses, we constructed Model A (Figure 1). Self-focused attention has been emphasized as being associated with cognitive maintaining factors in SAD (Clark and Wells, 1995; Rapee and Heimberg, 1997; Hofmann and Otto, 2008), but manipulation of self-focused attention has been reported to improve social anxiety (Bögels, 2006). Thus, we assumed a path from self-focused attention bias to social anxiety and constructed Model B (Figure 2) by adding that path to Model A. Furthermore, Calamaras et al. (2015) reported that probability bias has a direct positive impact on social anxiety. Therefore, we hypothesized a path from probability bias to social anxiety and constructed Model C (Figure 3) by adding that path to Model A. We did not assume error correlations in these models.

## Methods

### Participants and procedure

A questionnaire survey was conducted with 451 undergraduate students who study human sciences or computer sciences from three universities in Japan (January 2019–December 2019). We distributed the questionnaires, explained the ethical considerations in writing and verbally, and asked for the students’ consent to participate. The set of questionnaires consisted of the Japanese version of the Five Facet Mindfulness Questionnaire (Sugiura et al., 2012), the Japanese version of Self-Focused Attention Scale (Noda et al., 2021a), the Speech Cost/Probability Scale (Noda et al., 2017b), and the Japanese version of Liebowitz Social Anxiety Scale (Asakura et al., 2002) and was completed in the order listed to minimize the psychological burden in responding. Of the original sample, 367 participants (160 men, 204 women, and 3 who did not indicate their gender) completed all the scales, with an effective response rate of 81.37%. The participants’ ages ranged from 18 to 25 years, with a mean age of 19.92 years (*SD* = 0.88; two participants did not indicate their age). The demographic data of the participants are displayed in Table 1. This study was approved by the ethics committee of the first author’s affiliated university (approval number: 30021).

**TABLE 1:** demographic data.

	Participants (*N* = 367)	
Demographics	Frequency	Percentage
**Gender**		
Male	160	43.60%
Female	204	55.59%
Non-response	3	0.82%
**Age**		
18	9	2.45%
19	89	24.25%
20	218	59.40%
21	29	7.90%
22	14	3.81%
23	4	1.09%
24	1	0.27%
25	1	0.27%
Non-response	2	0.54%
**Universities**		
A university	173	47.14%
B university	151	41.14%
C university	43	11.72%
**Major**		
Human science	324	88.28%
Computer science	43	11.72%

### Measures

#### Japanese version of the five facet mindfulness questionnaire

The Five Facet Mindfulness Questionnaire (FFMQ) was developed by Baer et al. (2006) to measure trait mindfulness. It consists of five subscales: “observing,” “acting with awareness,” “non-judging,” “non-reactivity,” and “describing.” The scale includes 39 items, each rated on a 5-point scale from 1 (*never or very rarely true*) to 5 (*very often or always true*). Sugiura et al. (2012) developed the Japanese version of the FFMQ and demonstrated its reliability and validity. In this study, the total score of the five subscales was used as the score for trait mindfulness. The FFMQ scales showed acceptable internal consistency in the study (overall scale: Cronbach’s α = 0.82, observing: α = 0.74, acting with awareness: α = 0.83, non-judging: α = 0.86, non-reactivity: α = 0.68, and describing: α = 0.88).

#### Japanese version of self-focused attention scale

The Self-Focused Attention Scale (SFA) was developed by Bögels et al. (1996) to measure self-focused attention. The SFA comprises 11 items: 6 items for arousal and 5 items for behavior. Each item is rated on a 5-point scale from 0 (*not at all*) to 4 (*very much*). Noda et al. (2021a) developed the Japanese version of the SFA and confirmed its reliability and validity. The SFA showed good internal consistency in this study (Cronbach’s α = 0.90).

#### Speech cost/probability scale

The Speech Cost/Probability Scale (SCPS; Noda et al., 2017b) measures the cost/probability bias in speech situations where patients with SAD exhibit excessive anxiety. The scale requires the respondents to rate each item separately for cost bias and probability bias. The cost bias scale measures the degree of the cost bias by having participants rate statements such as, “when I speak in front of strangers, I think that I make mistakes and that strangers think of me as a stupid person,” and “when I feel that the audience is not listening to me, I think that my story is boring.” The probability bias scale requires the respondents to rate the expected likelihood of the occurrence of negative results for the same items. The scales consist of 11 items each, and each item is rated on a 5-point scale from 1 (*not at all* in the cost bias scale, and *I don’t think so at all* in the probability bias scale) to 3 (*very much* in the cost bias scale and *I very much think so* in the probability bias scale). The reliability and validity of the SCPS were confirmed by Noda et al. (2017b). The SCPS showed good internal consistency in the study (cost bias: Cronbach’s α = 0.90, probability bias: α = 0.88).

#### Liebowitz social anxiety scale self-reported version

The LSAS, developed by Liebowitz (1987), measures anxiety and avoidance behavior in 24 social situations. The scale consists of 24 items each for anxiety and avoidance behavior, and each item is rated on a 4-point scale from 0 (*none* on the anxiety scale and *never* on the avoidance behavior scale) to 3 (*severe* in the anxiety scale and *usually* on the avoidance behavior scale). The Japanese version of the LSAS was developed by Asakura et al. (2002) and has high reliability and validity (Asakura et al., 2002; Okajima et al., 2007). The LSAS showed good internal consistency in this study (overall scale: Cronbach’s α = 0.96, anxiety: α = 0.94, avoidance behavior: α = 0.92).

### Statistical analyses

First, to examine the relationships among trait mindfulness, self-focused attention, cost/probability bias, avoidance behavior, and social anxiety, Pearson’s correlation coefficients among each scale were computed. Second, path analyses were performed to examine the fit of the three hypothesized models, assuming that self-focused attention, cost/probability bias, and avoidance behavior mediated the relationship between trait mindfulness and social anxiety. Chi-square values (χ^2^), comparative fit index (CFI), Tucker–Lewis index (TLI), root mean square error of approximation (RMSEA), and standardized root mean squared residual (SRMR) were used as model fit indices. Good model fit was indicated by a non-significant χ^2^ value (Schermelleh-Engel et al., 2003). The CFI ranges from 0 to 1, with values closer to 1 indicating better fit. The closer the TLI is to 1, the better the fit; however, unlike the CFI, it may exceed 1. For CFI and TLI, values above 0.95 indicate a good fit and values above 0.97 indicate a very good fit (Schermelleh-Engel et al., 2003). In contrast, for RMSEA and SRMR, a value close to 0 is more appropriate. The RMSEA value of 0.05 or less is considered a good fit, 0.08 indicates an acceptable fit, and 0.10 or more is a poor fit (Browne and Cudeck, 1993). SRMR values below 0.08 are considered a good fit (Hu and Bentler, 1999). According to Ito (2018), the appropriate sample size for path analysis is 10 times or more the data of the free parameters. Since the free parameters of the hypothetical models are 22 and 23, we set the sample size at 230 or more.

SPSS version 24 (IBM Corp., Armonk, NY, United States) was used to compute the descriptive statistics and Pearson’s correlation coefficients, and path analyses and mediation analyses were performed using Mplus 8 (Muthén and Muthén, 1998–2017).

## Results

### Correlation analysis

Table 2 shows the correlation coefficients among all the variables of the study, as well as the descriptive statistics. As can be seen, the FFMQ total showed moderate negative correlations with the SFA (*r* = −0.37, *p* < 0.01), SCPS (cost bias: *r* = −0.44, *p* < 0.01; probability bias: *r* = −0.27, *p* < 0.01), and LSAS (total: *r* = −0.44, *p* < 0.01; anxiety: *r* = −0.49, *p* < 0.01; avoidance behavior: *r* = −0.33, *p* < 0.01).

**TABLE 2:** Descriptive statistics and correlations among the scales.

	Scales	Mean	SD	2	3	4	5	6	7	8	9	10	11	12
1	FFMQ total	113.60	14.55	0.12*	0.63**	0.60**	56**	0.73**	−0.37**	−0.44**	−0.27**	−0.44**	−0.49**	−0.33**
2	Observing	23.09	5.07	−	−0.26**	−0.39**	0.01	0.09	0.34**	0.20**	0.17**	0.04	0.05	0.04
3	Acting with awareness	24.47	5.67		−	0.40**	0.20**	0.24**	−0.33**	−0.29**	−0.20**	−0.23**	−0.28**	−0.15**
4	Non-judging	23.50	6.18			−	0.19**	0.25**	−0.47**	−0.37**	−0.22**	−0.27**	−0.31**	−0.20**
5	Non-reactivity	20.08	4.05				−	0.28**	−0.22**	−0.26**	−0.11*	−0.28**	−0.30**	−0.22**
6	Describing	22.46	6.08					−	−0.24**	−0.41**	−0.29**	−0.42**	−0.44**	−0.34**
7	SFA	23.26	9.54						−	0.59**	0.40**	0.43**	0.50**	0.31**
8	SCPS-cost bias	34.50	9.38							−	0.67**	0.64**	0.67**	0.52**
9	SCPS-probability bias	31.99	8.68								−	0.48**	0.47**	0.44**
10	LSAS total	61.89	28.96									−	0.94**	0.94**
11	Anxiety	34.12	15.78										−	0.77**
12	Avoidance behavior	27.77	15.02											−

**p < 0.01, *p < 0.05.

FFMQ, Five Facet Mindfulness Questionnaire; SFA, Self-Focused Attention Scale; LSAS, Liebowitz Social Anxiety Scale; SCPS, Speech Cost/Probability Bias Scale.

### Model testing

**FIGURE 4:**
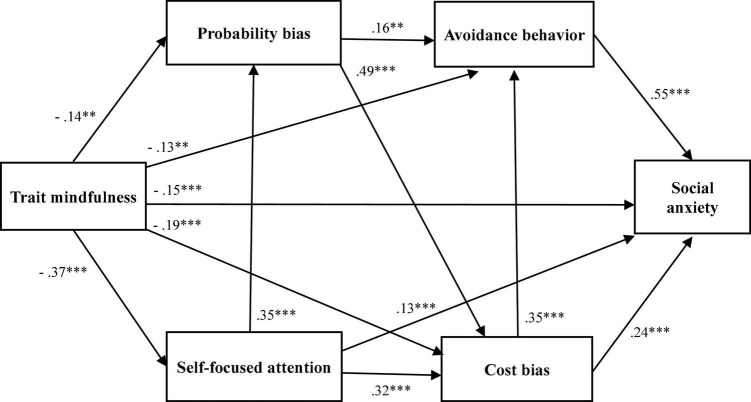
Model fit and standardized parameter estimates for Model B. Chi-square values, χ^2^ = 1.79, *df* = 2, *p* = 0.41; comparative fit index (CFI) = 1.000; Tucker–Lewis index (TLI) = 1.000; root mean square error of approximation (RMSEA) = 0.000, 90% CI = 0.000–0.100; and standardized root mean squared residual (SRMR) = 0.006, ^***^p < 0.001, ^**^p < 0.01.

Model A showed generally acceptable fit to the data (χ^2^ = 16.43, *df* = 3, *p* < 0.01, CFI = 0.987, TLI = 0.937, RMSEA = 0.110 [90% CI = 0.062–0.165], and SRMR = 0.016). All standardized parameter estimates were significant (*p* < 0.01). The standardized residuals between SFA and LSAS-anxiety were significant (*p* < 0.05); however, they were not significant among other variables. The normalized residuals for covariance between variables were not significant. Model B showed a good fit to the data (χ^2^ = 1.79, *df* = 2, *p* = 0.41, CFI = 1.000, TLI = 1.000, RMSEA < 0.001 [90% CI = 0.000–0.100], and SRMR = 0.006). All standardized parameter estimates were significant (*p* < 0.01). The standardized residuals and normalized residuals for covariance between variables were not significant. Model C showed generally insufficient fit to the data (χ^2^ = 15.00, *df* = 2, *p* < 0.01, CFI = 0.988, TLI = 0.908, RMSEA = 0.133 [90% CI = 0.076–0.200], and SRMR = 0.016). A standardized parameter estimate from probability bias to social anxiety was not significant (*p* = 0.23); but all others were significant (*p* < 0.01). The standardized residuals between SFA and LSAS-anxiety were significant (*p* < 0.05); however, they were not significant among other variables. The normalized residuals for covariance between variables were not significant. Table 3 shows the fit indices for both models. Model B was accepted because it had a good fit to the data, and no model modification index was detected (Figure 4). Model B showed that trait mindfulness was negatively associated with social anxiety *via* self-focused attention, cost bias, and avoidance behavior. In addition, we investigated the mediation effects of self-focused attention, cost bias, and avoidance behavior between trait mindfulness and social anxiety. To test the significance of mediation effects, we conducted mediation analyses using the bootstrapping method (N_bootstrap_ = 2,000), with social anxiety as the dependent variable; trait mindfulness as the independent variable; and self-focused attention, cost bias, and avoidance as mediating variables. The results showed significant point estimates, total effects, direct effects, and indirect effects for each variable (*p* < 0.01; Table 4).

**TABLE 3:** Fit indices for the models.

Model	χ^2^	df	CFI	TLI	RMSEA (90% [Cl])	SRMR
Model A	16.43**	3	0.987	0.937	0.110 (0.062–0.165)	0.016
Model B	1.79	2	1.000	1.000	0.000 (0.000–0.100)	0.006
Model C	15.00**	2	0.988	0.908	0.133 (0.076–0.200)	0.016

**p < 0.01.

CFI, comparative fit index; TLI, Tucker–Lewis index; RMSEA, root mean square error of approximation; 90% CI = 90% confidence interval; SRMR, standardized root mean squared residual.

**TABLE 4:** The mediation effects of self-focused attention, cost bias, and avoidance behavior between trait mindfulness and social anxiety.

	Parameter estimate	95% CI	Standardized parameter estimate	95% CI
**Self-focused attention**				
Point estimate from trait mindfulness to self-focused attention	−0.24**	[−0.31, −0.18]	−0.37**	[−0.46, −0.27]
Point estimate from self-focused attention to social anxiety	0.61**	[0.44, 76]	0.37**	[0.27, 46]
Total effect	−0.53**	[−0.63, −0.44]	−0.49**	[−0.57, −0.41]
Direct effect	−0.39**	[−0.49, −0.28]	−0.36**	[−0.45, −0.26]
Indirect effect	−0.15**	[−0.21, −0.10]	−	−
**Cost bias**				
Point estimate from trait mindfulness to cost bias	−0.29**	[−0.35, −0.23]	−0.44**	[−0.52, −0.37]
Point estimate from cost bias to social anxiety	0.95**	[0.82, 1.08]	0.57**	[0.48, 0.64]
Total effect	−0.53**	[−0.63, −0.44]	−0.49**	[−0.57, −0.41]
Direct effect	−0.26**	[−0.36, −0.17]	−0.24**	[−0.33, −0.16]
Indirect effect	−0.27**	[−0.34, −0.21]	−	−
**Avoidance behavior**				
Point estimate from trait mindfulness to avoidance behavior	−0.34**	[−0.44, −0.25]	−0.33**	[−0.42, −0.24]
Point estimate from avoidance behavior to social anxiety	0.71**	[0.63, 0.79]	0.68**	[0.61, 0.74]
Total effect	−0.53**	[−0.63, −0.44]	−0.49**	[−0.57, −0.41]
Direct effect	−0.29**	[−0.37, −0.22]	−0.27**	[−0.34, −0.20]
Indirect effect	−0.25**	[−0.32, −0.17]	−	−

**p < 0.01.

95% CI = 95% confidence interval.

## Discussion

The purpose of this study was to examine the relationships among trait mindfulness, self-focused attention, cost/probability bias, avoidance behavior, and social anxiety, and clarify whether these factors mediate the relationship between trait mindfulness and social anxiety. The results of the correlation analysis showed that trait mindfulness was negatively correlated with self-focused attention, cost bias, probability bias, avoidance behavior, and social anxiety. These results are consistent with previous studies (Schmertz et al., 2012; Noda et al., 2017a,2018a,b,2021a) and suggest that individuals with high trait mindfulness tend to have lower self-focused attention, cost bias, probability bias, avoidance behavior, and social anxiety.

The path analyses showed that Model B had a high degree of fit and satisfied all the criteria; therefore, Model B was accepted. Furthermore, the mediation analyses showed that self-focused attention, cost bias, and avoidance behaviors mediated the relationship between trait mindfulness and social anxiety. Since this study was cross-sectional in nature, causality could not be established. However, the model showed that trait mindfulness was negatively associated with social anxiety *via* self-focused attention, cost/probability bias, and avoidance behavior. From the path coefficients, it can be seen that trait mindfulness was the variable most highly and directly associated with self-focused attention among the variables analyzed in this study. Self-focused attention is a core factor in maintaining social anxiety (Clark and Wells, 1995; Rapee and Heimberg, 1997; Hofmann and Otto, 2008) and lower self-focused attention helps to prevent and treat social anxiety (Vriends et al., 2017). Baer (2009) indicated that increased trait mindfulness mediated the improvement in psychological functioning by cultivating an adaptive form of self-focused attention. It has also been reported that MBIs and MAGT reduce self-focused attention and social anxiety symptoms (Bögels et al., 2006; Desnoyers et al., 2017). Considering the results of this study, it is suggested that mindfulness training (MT) may reduce self-focused attention and contribute to the reduction of social anxiety.

The model shows that cost bias mediates the relationship between trait mindfulness and social anxiety, which is consistent with the findings of Schmertz et al. (2012). In addition, trait mindfulness was associated with cost bias through self-focused attention and probability bias. MBIs and MAGT have demonstrated efficacy in improving social anxiety as well as negative cognitions (Koszycki et al., 2007; Piet et al., 2010; Goldin et al., 2016). Based on the results of this study, it is speculated that MT may ameliorate cost bias and consequently reduce social anxiety.

It was also shown that avoidance behavior mediates the relationship between trait mindfulness and social anxiety, which supports the findings of Noda et al. (2017a). Moreover, trait mindfulness was associated with avoidance behavior through probability bias and cost bias. Among the variables treated in this study, the coefficient of the path from avoidance behavior to social anxiety was found to be the highest. Avoidance behavior is a maintaining factor of social anxiety, and individuals with high social anxiety engage in more avoidance behaviors (Clark and Wells, 1995; McManus et al., 2008). Therefore, managing avoidance behavior is a key component in the treatment of social anxiety (Clark and Wells, 1995; Rapee and Heimberg, 1997; Hofmann and Otto, 2008). MBIs and MAGT have been found to be effective in reducing avoidance behavior and social anxiety (Koszycki et al., 2007; Goldin et al., 2016; Desnoyers et al., 2017). Additionally, it is suggested that MT may reduce avoidance behavior and contribute to the amelioration of social anxiety.

Previous CBT treatments for social anxiety are based on the cognitive behavioral models (Clark and Wells, 1995; Rapee and Heimberg, 1997; Hofmann and Otto, 2008), combining intervention techniques for each maintaining factor of social anxiety. The MAGT program is also structured based on a cognitive behavioral model (Fleming and Kocovski, 2007). The findings of the present study may be useful in constructing MBIs and MAGT programs for social anxiety. The results indicated that trait mindfulness was associated with social anxiety directly, as well as through self-focused attention, cost/probability bias, and avoidance behavior. MT, as an intervention technique, may improve not only social anxiety but also its maintaining factors (Koszycki et al., 2007; Piet et al., 2010; Kocovski et al., 2013; Goldin et al., 2016; Desnoyers et al., 2017). Therefore, it is expected that MT might be an effective intervention technique for SAD and individuals with high social anxiety. In addition, the present study showed that avoidance behavior was strongly associated with social anxiety. Exposure is a CBT technique that targets avoidance behavior as a treatment. England et al. (2012) reported that the combination of MT and exposure was more effective than exposure with a habituation rationale in helping patients with SAD achieve diagnostic remission. These findings suggest that the combination of MT and CBT techniques may be used for reducing avoidance behavior, as it may have a greater effect on improving social anxiety.

This study provides impetus for future research on clinical studies of MBIs and MAGT for social anxiety. MBIs and MAGT have been previously shown to be less effective than CBT (Liu et al., 2021). However, it has been suggested that MT may enhance the therapeutic effect of CBT (Barlow et al., 2011; Shirotsuki and Noda, 2018; Noda et al., 2021b). Based on this model, it is possible that an MAGT and MBIs program that combines MT and CBT techniques may show better effectiveness.

## Limitations

Several limitations of this study must be considered. First, further investigation will be necessary for patients with SAD as this study only investigated university students. The average LSAS score of the participants in this study was 61.89. The score exceeds the cut-off value of 44 points for clinical groups, suggesting that many of the participants had clinical symptoms of SAD. It has been suggested that SAD patients and the general population fall on a continuum of social anxiety symptoms (McNeil, 2010; Ruscio, 2010). Thus, it is useful to analyze the present study sample to examine the psychological characteristics of SAD. However, in the present study, patients with SAD were not included. In the future, it is necessary to examine the psychological mechanisms that cause self-focused attention, cost/probability bias, and avoidance behavior to mediate the relationship between trait mindfulness and social anxiety in patients with SAD, and to clarify the possibility of applying it to a clinical group.

Second, although the models for this study were constructed based on previous studies, assuming directions of causality, as the present study used a cross-sectional design, causal relationships between the variables cannot be established based on the present results. In the future, it is necessary to identify detailed causal relationships between the variables through intervention studies using MT.

Third, further testing of the model of mindfulness affecting social anxiety is needed. Error correlations were not evaluated in this study because Model B’s goodness-of-fit indices were high and no model modification index was detected. However, error correlations may exist in this model since correlation coefficients among variables are relatively high. Furthermore, in addition to the variables addressed in this study, there are other factors that contribute to the maintenance of SAD, such as safety behaviors and social skills. Considering the limitations above, it is necessary to construct a model in the future in which mindfulness affects social anxiety and to examine its validity.

## Conclusion

The present study revealed the complex relationships among trait mindfulness, self-focused attention, cost/probability bias, avoidance behavior, and social anxiety. These findings indicate that mindfulness plays an important role in social anxiety and contribute to future research involving clinical studies of interventions aimed at enhancing mindfulness, such as MBIs and MAGT.

## Data availability statement

Detailed data are available from the corresponding author upon reasonable request.

## Ethics statement

The studies involving human participants were reviewed and approved by Musashino University Human Sciences Department Ethics Committee. Written informed consent for participation was not required for this study in accordance with the national legislation and the institutional requirements.

## Author contributions

SN contributed to the study conception and design, performed material preparation, data collection, and analysis and wrote the first draft of the manuscript. All authors commented on previous versions of the manuscript and read and approved the final manuscript.
